# Emergence of Ceftazidime/Avibactam and Tigecycline Resistance in Carbapenem-Resistant *Klebsiella pneumoniae* Due to In-Host Microevolution

**DOI:** 10.3389/fcimb.2021.757470

**Published:** 2021-10-25

**Authors:** Xinhong Han, Qiucheng Shi, Yihan Mao, Jingjing Quan, Ping Zhang, Peng Lan, Yan Jiang, Dongdong Zhao, Xueqing Wu, Xiaoting Hua, Yunsong Yu

**Affiliations:** ^1^ Department of Infectious Diseases, Sir Run Run Shaw Hospital, School of Medicine, Zhejiang University, Hangzhou, China; ^2^ Key Laboratory of Microbial Technology and Bioinformatics of Zhejiang Province, Hangzhou, China; ^3^ Regional Medical Center for National Institute of Respiratory Diseases, Sir Run Run Shaw Hospital, Zhejiang University School of Medicine, Hangzhou, China; ^4^ Department of Clinical Laboratory, The Children’s Hospital, Zhejiang University School of Medicine, National Clinical Research Center for Child Health, Hangzhou, China

**Keywords:** carbapenem-resistant *K. pneumoniae*, ceftazidime/avibactam, tigecycline, *in vivo*, insertion sequences

## Abstract

*Klebsiella pneumoniae* can cause both hospital- and community-acquired clinical infections. Last-line antibiotics against carbapenem-resistant *K. pneumoniae* (CRKP), such as ceftazidime/avibactam (CZA) and tigecycline (TGC), remain limited as treatment choices. This study aimed to investigate the mechanisms by which CRKP acquires CZA and TGC resistance *in vivo* under β-lactam antibiotic and TGC exposure. Three CRKP strains (XDX16, XDX31 and XDX51) were consecutively isolated from an inpatient with a urinary tract infection in two months. PFGE and MLST showed that these strains were closely related and belonged to sequence type (ST) 4496, which is a novel ST closely related to ST11. Compared to XDX16 and XDX31, XDX51 developed CZA and TGC resistance. Sequencing showed that double copies of *bla*_KPC-2_ were located on a 108 kb IncFII plasmid, increasing *bla*_KPC-2_ expression in XDX51. In addition, *ramR* was interrupted by Insertion sequence (IS) Kpn14 in XDX51, with this strain exhibiting upregulation of *ramA*, *acrA* and *acrB* expression compared with XDX16 and XDX31. Furthermore, LPS analysis suggested that the O-antigen in XDX51 was defective due to IS*Kpn26* insertion in the rhamnosyl transferase gene *wbbL*, which slightly reduced TGC susceptibility. In brief, CZA resistance was caused mainly by *bla*_KPC-2_ duplication, and TGC resistance was caused by *ramR* inactivation with additional LPS changes due to IS element insertion in *wbbL*. Notably, CRKP developed TGC and CZA resistance within one month under TGC and β-lactam treatment without exposure to CZA. The CRKP clone ST4496 has the ability to evolve CZA and TGC resistance rapidly, posing a potential threat to inpatients during antibiotic treatment.

## Introduction

*Klebsiella pneumoniae*, is a major pathogen that can cause both nosocomial- and community-acquired infections, such as urinary tract infections, bacteremia, respiratory infections and soft tissue infections, especially in immunocompromised individuals (Ullmann, 1998; Holt et al., 2015). The emergence of carbapenem-resistant *K. pneumoniae* (CRKP) has limited antibiotic therapeutic choices and led to serious challenges in clinical treatment and infection control (Rice, 2008; Livermore et al., 2015a; Moradigaravand et al., 2017). The World Health Organization (WHO) considers carbapenem-resistant *Enterobacteriaceae* to be critical priority pathogens that require new antibiotics (Shrivastava et al., 2018). Ceftazidime/avibactam (CZA) and tigecycline (TGC) are last-line choices for the treatment of CRKP infections.

CZA is a novel β-lactam-β-lactamase inhibitor combination with the ability to inhibit the activity of AmpC, extended-spectrum β-lactamases (ESBLs), class A carbapenemases such as KPC and class D carbapenemases such as OXA-48 but not class B carbapenemases such as NDM, IMP and VIM (Shirley, 2018). CZA was approved for the treatment of complicated intra-abdominal infections and urinary tract infections by the FDA on February 25, 2015. A multicenter, observational study reported that CZA could be a reasonable substitute for colistin for the treatment of CRKP infections with reduced mortality (Van Duin et al., 2018). In the International Network For Optimal Resistance Monitoring (INFORM) surveillance programme, the *in vitro* susceptibility of carbapenemase-positive and MBL-negative isolates to CZA was 99.8% between 2015 and 2017 (Spiliopoulou et al., 2020). However, acquisition of CZA resistance has been reported after CZA therapy in recent years. Resistance to CZA has been observed in strains with mutations in AmpC, *bla*_KPC-2_ and *bla*_KPC-3_, and the mutation point was mostly in Ω-loop in *bla*_KPC_ genes (Shirley, 2018; Zhang et al., 2020). In addition, high expression of KPC-3 was reported to be associated with CZA resistance (Humphries and Hemarajata, 2017).

The emergence of TGC resistance after exposure to TGC has also been reported (Duin et al., 2014; Lin et al., 2016; Ye et al., 2017). Overall, the primary TGC resistance mechanisms in *K. pneumoniae* include overexpression of efflux pumps, such as AcrAB and OqxAB, which can be mediated by regulators (RamR-RamA and OqxR-RarA) and global activators (MarA, SoxS and Rob) (Veleba and Schneiders, 2012). There also exist other known mechanisms, conferred by the Lon protease, Tet(A) protein and ribosomal protein S10, which is encoded by *rpsJ* (Villa et al., 2014). In addition, Linkevicius M et al. reported that lipopolysaccharide (LPS) defects could lead to reduced susceptibility to TGC (Linkevicius et al., 2016).

Among all the antibiotics tested by the INFORM surveillance programme (2015–2017) against 1,460 carbapenem-nonsusceptible *Enterobacteriaceae* isolates, CZA and TGC showed the highest susceptibility rates (73.0% and 78.1%, respectively) (Spiliopoulou et al., 2020). Strains resistant to these last-line antibiotics could pose a great threat to public health.

Here, we report a series of CRKP strains isolated in 2017 that have evolved resistance to both CZA and TGC during exposure to β-lactam antibiotics and TGC. The CRKP isolates resistant to CZA were isolated before CZA was approved in China. The aim of this study was to describe the phenotypic and genotypic adaption characteristics of these strains during in-host evolution and explain the molecular mechanisms of CZA and TGC resistance.

## Material and Methods

### Strains and Patient Characteristics

**Figure 1:**
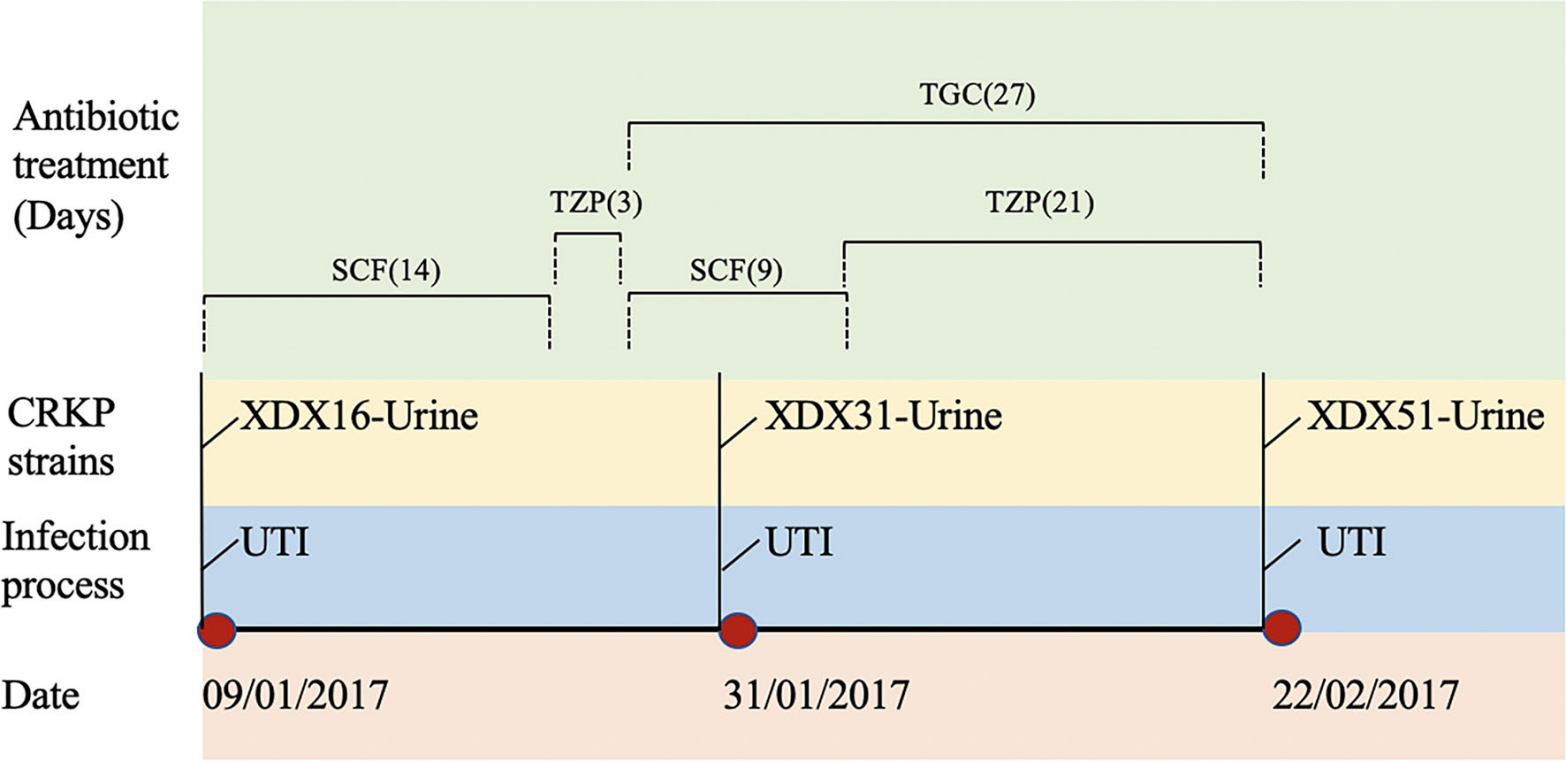
Timeline of CRKP infection and treatment during the patient’s hospitalization. XDX16, XDX31 and XDX51 are carbapenem-resistant *Klebsiella pneumoniae* strains. UTI, urinary tract infection; SCF, Cefoperazone-sulbactam; TZP, Piperacillin-tazobactam; TGC, Tigecycline.

Three CRKP strains (XDX16, XDX31 and XDX51) were isolated from an 85-year-old male patient hospitalized in Sir Run Run Shaw Hospital, Zhejiang Province, China, in 2017. The patient was admitted for a urinary tract infection with multiple organ dysfunction. After admission, he received urological surgeries, including partial cystectomy, prostatic enucleation surgery and ureteric reimplantation. The patient suffered urinary tract infection with prolonged fever. Piperacillin-tazobactam (TZP, 4.5 g every 8 h) and cefoperazone-sulbactam (SCF, 2 g every 8 h) were alternately used as empirical therapies. Then, TGC (50 mg every 12 h) was used as a definitive therapy. During this period, three CRKP strains, named XDX16, XDX31 and XDX51, were isolated from the patient’s urine. In addition, XDX51 exhibited resistance to both CZA and TGC. The patient chose to be discharged against medical advice. The timeline of CRKP infection and antibiotic usage was shown in **Figure 1**. This study was approved by the ethics committees of the participating hospital (20170301-3)

### Antimicrobial Susceptibility Testing

The antibiotics tested in this study were TGC, CZA, meropenem, imipenem, ertapenem, ceftazidime, amikacin, levofloxacin, fosfomycin, aztreonam and colistin. The antimicrobial susceptibility tests and minimum inhibitory concentrations (MICs) were based on the European Committee on Antimicrobial Susceptibility Testing (EUCAST, Version 10.0, 2020) guidelines and breakpoints. The MICs of carbapenem and fosfomycin (with supplementation of glucose-6-phosphate in the agar) were determined by the agar dilution method, and the others were determined by the broth microdilution method with fresh cation-adjusted Mueller-Hinton broth. The MICs of TGC were also determined in the presence of Phe-Arg-β-naphthylamide (PAβN) at a concentration of 50 μg/mL to verify the function of the efflux pump in TGC resistance (Salehi et al., 2021).

### Whole-Genome Sequencing and Analysis

The clonality of the strain series was confirmed by pulsed-field gel electrophoresis (PFGE) with a previously described protocol (Barton et al., 1995). Briefly, genomic DNA was digested with the restriction endonuclease XbaI and electrophoresed at 14°C for 20 h.

XDX16 and XDX51 were subjected to both Illumina paired-end sequencing (Illumina Inc., San Diego, CA) and long-read nanopore sequencing (Oxford Nanopore Technologies, Oxford, UK). For Illumina sequencing, genomic DNA was extracted using a QIAamp DNA MiniKit (Qiagen, New York, USA). The whole genome was assembled by canu (Koren et al., 2017), and the *de novo* assemblies were subsequently annotated with the Prokka pipeline (Seemann, 2014). Resistance genes were detected by ResFinder 3.2 with a 90% threshold for gene identification and a 60% minimum length coverage (Zankari et al., 2012). Multilocus sequence typing (MLST) was performed with the Center for Genomic Epidemiology guidelines (http://cge.cbs.dtu.dk/services/MLST/). Breseq (version 0.27.1) was used to find mutations in XDX51, with XDX16 as the reference strain (Barrick and Deatherage, 2014). All the mutations in XDX16, XDX31 and XDX51 were confirmed by PCR and Sanger sequencing (the primers are listed in **Table S1**).

The nucleotide sequences of XDX16 and XDX51 have been submitted to the NCBI database under the accession JAIWPV000000000-JAIWPW000000000.

### qRT-PCR for Gene Expression Analysis

qRT-PCR was used to measure the expression of *bla*_KPC-2_, *acrA*, *acrB* and *ramR* of XDX16, XDX31 and XDX51. XDX16 was used as the reference strain, and the *rpoB* gene was used as the internal reference (the primers are listed in **Table S1**). RNA was extracted using the PureLink RNA Mini Kit (Invitrogen, Carlsbad, CA) in the exponential growth period of bacterial cells. Then, cDNA was obtained using a PrimeScript™ RT Reagent Kit (Takara, Kyoto, Japan). The expression level was assessed using TB Green™ Premix Ex Taq™ (Takara, Kyoto, Japan) in a LightCycler 480 system (Roche, Rotkreuz, Switzerland) with triplicate samples for each isolate, replicating three times independently using the comparative C_T_ method. Genes were considered to be differentially expressed when the |log_2_ fold change| was greater than 1.5 (Livak and Schmittgen, 2001). The log2 fold change in *bla*_KPC-2_ expression was analyzed by an unpaired t test on GraphPad Prism, and *p*<0.05 was considered significant.

### Gene Knockout and Complementation Experiment

We used the lambda-Red knockout system described previously for *wbbL* gene knockout (Huang et al., 2014). Briefly, the FRT-flanked apramycin resistance cassette was amplified from the pIJ773 plasmid using a homologous region primer. The pACBSR-Hyg plasmid was introduced into XDX16 by electroporation for recombination. The knockout cassette was transformed into XDX16-pACBSR-Hyg. Correct transformants were screened using LBApra at 37°C and verified by PCR and Sanger sequencing. Colonies were screened for the loss of pACBSR-Hyg by streaking onto LBApra with or without hygromycin (100 μg/mL) and low-salt LB + hygromycin plates at 37°C overnight. The resistance marker was removed by pFLP-Hyg plasmid.

The amplified target gene (*wbbL*) and its promoter were cloned into the pCR2.1-Hyg plasmid, which was digested with the Xba1 FastDigest enzyme (Thermo Scientific, Waltham, the USA). The recombinant plasmids were introduced to resistant and knockout strains (XDX51 and XDX16∆*wbbL*) *via* chemical transformation. The empty vector (pCR2.1-Hyg) was introduced to the same strains as a blank control. The complemented sequence was confirmed by Sanger sequencing (the primers are listed in **Table S1**).

### LPS Analysis

Extraction of XDX16, XDX16∆*wbbL* and XDX51 LPS was performed according to Michael R. Davis’s protocol (Davis and Goldberg, 2012). Then, 5 μl of LPS was electrophoresed on 15% Tris-glycine (Fdbio Science, Hangzhou, China) and directly stained using a fast silver staining kit (Beyotime Biotechnology, Shanghai, China). O-antigen typing analysis was performed using Kaptive (http://kaptive.holtlab.net/).

## Results

### Strains and Antibiotic Susceptibility

XDX16 and XDX31 were considered the index strains. Three strains were all identified to be resistant to meropenem, imipenem, ertapenem, ceftazidime, amikacin, levofloxacin and aztreonam. Significant differences in MICs were observed for CZA and TGC between XDX51 and the index strains. Specifically, for the index strains, TGC had an MIC of 0.5 μg/mL, while for XDX51, the MIC of TGC was 8-fold higher. Additionally, the MIC of CZA for XDX51 (16 μg/mL) was 4-fold higher than that for the index strains (4 μg/mL). In addition, the MICs of meropenem, imipenem and ceftazidime for XDX51 were also higher than those for the index strains (**Table 1**).

**Table 1:** Antimicrobial susceptibility of isolates used in the study.

Strains	MIC (μg/mL)*											
	TGC	MEM	IMP	ETP	CAZ	AK	LEV	CZA(4)	CZA(8)	CST	FOS	ATM
XDX16	0.5	128	64	>128	64	>512	>8	4	2	0.06	32	>64
XDX31	0.5	128	64	>128	64	>512	>8	4	2	0.06	32	>64
XDX51	4	>128	128	>128	512	>512	>8	16	4	0.03	32	>64
XDX51::pCR2.1-Hyg^a^	4	>128	128	>128	>128	>512	>8	16	–	0.03	32	>64
XDX51::p*wbbL* ^b^	2	>128	128	>128	>128	>512	>8	16	–	0.03	32	>64
XDX16△*wbbL* ^c^	1	128	64	>128	64	>512	>8	4	–	0.06	32	>64
XDX16△*wbbL*::pCR2.1-Hyg^d^	1	128	64	>128	64	>512	>8	4	–	0.06	32	>64
XDX16△*wbbL*::p*wbbL* ^e^	0.5	128	64	>128	64	>512	>8	4	–	0.06	32	>64
XDX51+PAβN	1	–	–	–	–	–	–	–	–	–	–	–

*TGC, tigecycline; MEM, meropenem; IPM, imipenem; ETP, ertapenem; CAZ, ceftazidime; AK, amikacin; LEV, levofloxacin; CZA, ceftazidime/avibactam; CST, colistin; FOS, fosfomycin; ATM, aztreonam. ^a^pCR2.1-Hyg was constructed via TA clone with hyg connected to pCR2.1 plasmid. XDX51:: pCR2.1-Hyg indicated pCR2.1-Hyg plasmid was introduced into XDX51 by electrotransformation as blank control. ^b^XDX51::pwbbL indicated wild-type wbbL bearing pCR2.1-Hyg plasmid was introduced into XDX51 by electrotransformation. ^C^XDX16△wbbL was a wbbL knockout XDX16 strain. ^d^XDX16△wbbL::pCR2.1-Hyg indicated pCR2.1-Hyg plasmid was introduced into XDX16△wbbL as control. ^e^XDX16△wbbL::pwbbL indicated wild-type wbbL bearing pCR2.1-Hyg plasmid was introduced into XDX16△wbbL. CZA (4): The MIC of CZA was tested under 4 mg/L avibactam; CZA (8): The MIC of CZA was tested under 8 μg/mL avibactam.

### Genomic Characteristics

The PFGE results confirmed that the three strains were closely related (**Figure S1**). XDX16 and XDX51 were chosen for whole-genomic sequencing since XDX31 seemed to be the same passage as XDX16, with which it shared an identical PFGE typing result and resistance phenotype. According to genome-based MLST analysis, XDX16 and XDX51 belonged to the same novel ST, ST4496, which is closely related to ST11 with one single-locus variant on *mdh*.

XDX16 and XDX51 shared the same resistance gene distribution, including the aminoglycoside resistance genes *aadA2* and *rmtB*, the sulfonamide resistance gene *sul1*, the beta-lactam resistance genes *bla*_KPC-2_ and *bla*_SHV-182_, the fosfomycin resistance gene *fosA* and the quinolone resistance genes *oqxA* and *oqxB*. Both of them had identical mutations in *acrR* and *ompK35*.

### Identification of Plasmid Differences in XDX51

**Figure 2:**
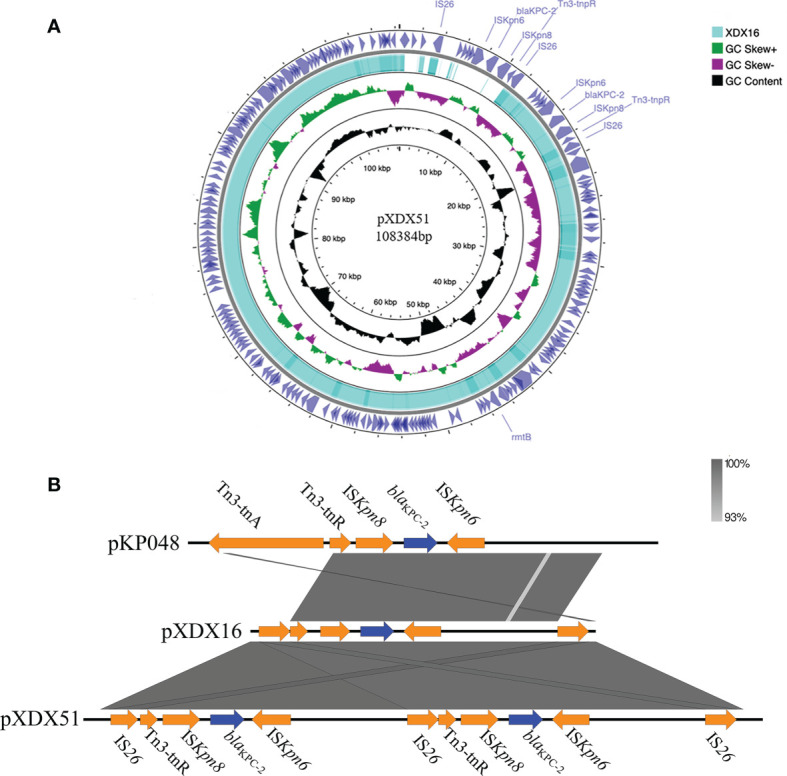
Characteristics of the *bla*_KPC-2_-bearing IncFII plasmid and the genetic environment of *bla*_KPC-2_. **(A)** Comparison of the IncFII plasmids in XDX51 and XDX16. There were double copies of *bla*_KPC-2_ in XDX51 compared to XDX16. **(B)** The *bla*_KPC-2_ environment in our study was compared with that in pKP048. The gene environment of *bla*_KPC-2_ in our study was IS*26*-Tn3-IS*Kpn8*-*bla*_KPC-2_-IS*Kpn6*-hypothetical protein-IS*26*. Blue arrows indicate resistance genes, and orange arrows indicate insertion sequences. The right inverted repeat (IRR) of IS*26* was 5’-ggcactgttgcaaa-3’, the left inverted repeat (IRL) of IS*26* was 5’-tttgcaacagtgcc-3’; the IRR of IS*Kpn8* was 5’-atgtcaagacccggctggttat-3’; and the IRL of IS*Kpn8* was 5’-atcccacgagtccagac-3’.

Both XDX16 and XDX51 had the IncFII plasmid harboring the antibiotic resistance genes *bla*_KPC-2_ and *rmtB*. Furthermore, there was duplication of *bla*_KPC-2_ on the plasmid of XDX51 (**Figure 2A**). The two copies were linked together and shared the same Insertion sequence (IS) 26. The surrounding structure of *bla*_KPC-2_ in our study was similar to the classic *bla*_KPC-2_ genetic environment in pKP048 in China reported in a previous study, with the gene order IS*26*, Tn3-resolvase, IS*Kpn8*, *bla*_KPC-2_, IS*Kpn6*, hypothetical protein and IS*26* (Shen et al., 2009) (**Figure 2B**). In our study, there were two IS*26* elements around the Tn3-based *bla*_KPC-2_ structure, with one IS*26* replacing the Tn3 transposase with pKP048.

### Identification of Whole-Genome Differences in XDX51

**Figure 3:**
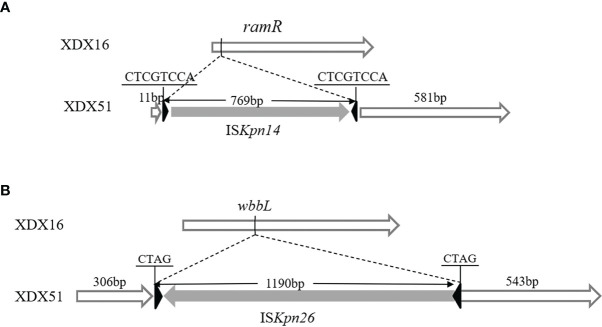
Schematic diagram of the gene structure. **(A)** The *ramR* gene was interrupted by IS*Kpn14* in XDX51; the IRL of IS*Kpn14* was 5’-GGTAATG-3’, and the IRR was 5’-CATTACC-3’. **(B)** The *wbbL* gene was interrupted by IS*Kpn26* in XDX51, the IRL of IS*Kpn26* was 5’-GGAAGGTGCGAA-3’, and the IRR was 5’-TTCGCACCTTCC-3’.

Comparison of the whole genomes of XDX16 and XDX51 also revealed that the *wbbL* gene (a rhamnosyl transferase gene) and *ramR* gene were interrupted by insertion sequences (**Figure 3**). A 769 bp IS*Kpn14* sequence was inserted at the 12 bp of *ramR* in XDX51. The insertion was responsible for interruption of the translation of the N-terminal amino acid sequence of RamR in XDX51. Similarly, *wbbL* was interrupted by a 1190 bp IS*Kpn26* sequence at 307 bp in XDX51. There were three SNPs detected in XDX51 compared to the prior wild type XDX16 using breseq. However, the three SNPs have occurred in the next wild type XDX31 isolate without TGC and CZA resistance phenotype. Hence, we inferred that these three SNPs were not responsible for TGC and CZA resistance (**Table S2**).

### Expression Level of Antibiotic Resistance Genes

**Figure 4:**
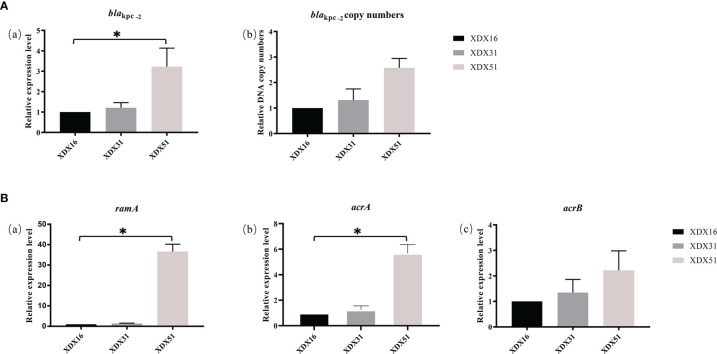
Relative expression level or DNA copy numbers of **(A)** *bla*_KPC-2-_ and **(B)** AcrAB-TolC-related genes. XDX16 was used as the reference strain, and *rpoB* was used as the reference gene. The bars represent the mean ± standard deviation (SD) of triplicate biological repeats; the mean differences in log_2_ fold change were analyzed using an unpaired t test. *p < 0.05.

Compared to the copy numbers in XDX16, the copy numbers of *bla*_KPC-2_ in XDX51 determined by qRT-PCR (2.57 ± 0.3) were consistent with those found in the genome sequence. The *bla*_KPC-2_ expression level of XDX51 was 3.54 ± 0.5 times higher than that of XDX16 (**Figure 4A**). To verify the function of enhanced KPC expression on CZA resistance in XDX51, we performed CZA MIC test with avibactam at a higher concentration of 8 μg/mL, after which XDX51 could restore its susceptibility to CZA under sufficient avibactam.

*ramR* is a regulator of the AcrA/B system. We evaluated the regulatory effects of *ramR* alteration in XDX51 by qRT-PCR. The results showed that insertion in *ramR* led to overexpression of *ramA*, which was 36.6 times higher in XDX51 than in XDX16. The *acrA* and *acrB* expression levels in XDX51 were also upregulated compared to those in the index strains (**Figure 4B**). These results suggested that insertion in *ramR* affected the expression level of AcrAB-TolC efflux pumps. To further confirm the effect of *ramR* alteration on efflux pumps, we performed an efflux pump suppression test with PAβN, and there was a 4-fold decrease in the MIC of TGC for XDX51.

### Effect of the *wbbL* Gene on Phenotype

To further verify the effect of *wbbL* inactivation on antibiotic resistance, we constructed *wbbL* gene knockout and complementation strains. The MIC of TGC for the XDX16 *wbbL* gene knockout strain (XDX16∆*wbbL*) (1 μg/mL) increased 2-fold compared with that for XDX16 (0.5 μg/mL). The pCR2.1-Hyg vector was transformed into XDX16∆*wbbL* and into XDX51 as a control. Wild-type *wbbL* was cloned into the pCR2.1-Hyg vector (p*wbbL*) and introduced to the gene knockout strain (XDX16∆*wbbL*) and TGC-resistant strain with inactivated *wbbL* (XDX51). TGC sensitivity was restored in XDX16∆*wbbL*::p*wbbL* (0.5 μg/mL) and decreased in XDX51::p*wbbL* (2 μg/mL) but not in the empty vector-harboring strain. These results suggested that *wbbL* had a slight effect on the TGC resistance phenotype. The MICs of the isolates are presented in **Table 1**.

*wbbL* belongs to the O-antigen cluster. We hypothesized that *wbbL* could affect LPS biosynthesis in XDX51. Thus, LPS in XDX16∆*wbbL* and XDX51 was analyzed by SDS-PAGE and silver staining. Generally, the O-antigen bands were within 15–40 kD. There were fewer bands in XDX16∆*wbbL* and XDX51 within the 35–40 kD range, indicating that the O-antigen in XDX51 was shorter than that in the *wbbL* wild-type strain XDX16 (**Figure S2**).

## Discussion

Under antibiotic pressure, bacteria adapt to the host or environment with genomic and phenotypic changes (Linkevicius et al., 2016). Here, we described a series of closely related CRKP strains that evolved resistance to CZA and TGC *in vivo* in a hospitalized patient. ST analysis showed that the strains belonged to a new ST, ST4496, closely related to ST11. Before the CZA- and TGC-resistant strain XDX51 was isolated, TGC was used for 27 days, and β-lactam antibiotics (cefoperazone-sulbactam and piperacillin-tazobactam) were prescribed alternately, while there was no exposure to CZA. Compared to the index strains, three genomic differences were identified in XDX51, including duplication of *bla*_KPC-2_, *ramR* and *wbbL* insertions caused by IS elements. Ye et al. reported that deletion of the *ramR* ribosomal binding site could cause *in vivo* development of TGC resistance during TGC treatment (Ye et al., 2017). Long-term usage of antibiotics such as β-lactams and TGC could have led to the evolution of the series of CRKP strains in our study with gene mutations related to antibiotic resistance.

Increased KPC expression could enhance ceftazidime hydrolysis, which could not be completely inhibited by avibactam (Zhang et al., 2020). Both XDX16 and XDX51 carried an IncFII plasmid containing *bla*_KPC-2_ and *rmtB*. The MICs of CZA were 4-fold higher for XDX51 than for XDX16. There was duplication of *bla*_KPC-2_ on the XDX51 plasmid due to unequal crossover of the IS*26* composite transposon causing enhanced KPC expression levels, as identified by qRT-PCR. KPC-23-producing *K. pneumoniae* has been reported to be resistant to CZA due to increased ceftazidime hydrolysis without prior exposure to CZA (Galani et al., 2019). Similarly, Humphries RM reported CZA resistance due to increased expression of KPC-3 in clinical isolates previously unexposed to CZA (Humphries and Hemarajata, 2017). Both situations above were combined with membrane porin deficiency. In addition, Ω-loop alterations caused by KPC mutations can prevent avibactam binding and lead to CZA resistance, such as mutations at position 179 in *bla*_KPC-2_ or *bla*_KPC-3_ (Livermore et al., 2015b; Barnes et al., 2017). CZA resistance caused by KPC point mutations tends to occur after the strains are exposed to CZA either *in vivo* or *in vitro* (Livermore et al., 2015b; Barnes et al., 2017; Shields et al., 2017). Additionally, Antinori E. reported that deletion in *bla*_KPC-3_ in clinical *K. pneumoniae* could also result in CZA resistance (Antinori et al., 2020). In our study, the CZA-resistant strain evolved double copies of *bla*_KPC-2_ compared with the index strains. An increase in the MICs of carbapenem and CAZ for XDX51 indicated enhanced CAZ hydrolysis due to overexpression of *bla*_KPC-2_. Our conclusion was confirmed by other studies as well. Shen Z. et al. reported that the expression level of *bla*_KPC-2_ in CRKP with CZA MIC 4-8 μg/mL group was 4.2-4.8-fold higher than that in CZA MIC 1-2 μg/mL and ≤0.5 μg/mL group, and hydrolysis activities of CAZ was 4-4.6-fold higher in CZA MIC 4-8 group than the other two groups, indicating that the enhanced expression of *bla*_KPC-2_ could result in the decrease of CZA susceptibility due to the increased hydrolysis activity of CAZ (Shen et al., 2017). Hence, we considered the enhanced KPC expression was responsible for CZA resistance in XDX51.

Nonfunctional RamR can lead to TGC resistance *via* regulation of efflux pumps. Mutations in *ramR*, including deletion, mutation and insertion, resulting in TGC resistance have been previously reported (Hentschke et al., 2010; Ye et al., 2017). In XDX51, *ramR* insertion caused by IS*Kpn14* was identified through whole-genome sequencing. It was interrupted at 12 bp, affecting the translation of N-terminal amino acids. The RamR N-terminus acts as a DNA-binding site (Yamasaki et al., 2013). Without its DNA binding function, RamR is not able to inhibit RamA expression, and as a result, the efflux pump AcrAB-TolC is upregulated. As we speculated, qRT-PCR showed upregulated expression levels of *ramA*, *acrA* and *acrB* compared to that in the baseline strains. Moreover, efflux pump inhibition experiments with PA*β*N demonstrated the role of AcrAB-TolC in the increase in the TGC MIC in XDX51. Briefly, in our study, the dysregulation of RamR caused by IS element insertion was a major molecular mechanism for TGC resistance in XDX51.

In addition to antibiotic resistance-related genomic differences, LPS phenotype-related gene mutations occurred during within-host evolution. The 307 bp *wbbL* gene in XDX51 was interrupted by IS*Kpn26*, which shares 99% amino acid similarity with IS*5*. WbbL is a rhamnosyl transferase that transfers L-Rha residues to the O4 position of D-Glc or D-GlcNAc to obtain a complete O-antigen (Erbing et al., 1977; Izquierdo et al., 2003). O-antigen typing showed that XDX16 and XDX51 belonged to OL101 and that *wbbL* was an important component of the OL101 locus (Follador et al., 2016). LPS analysis suggested that the O-antigen in the XDX51 and *wbbL* knockout strains was shorter than that in the *wbbL* wild-type strain. In addition, the TGC MIC for the reconstructed *wbbL* defective mutant was 2-fold higher than that for the strain with the complete *wbbl* sequence. Leth K et al. reported that *wbbL* mutations could cause serotype shifts and MIC changes for mecillinam in combination with *bla*_CTX-M_ mutations in *E. coli.* A similar study also reported that mutations in LPS-related genes could cause antibiotic resistance (Anton, 1995; Leth et al., 2019). In our study, the *wbbL* mutation had a slight effect on TGC susceptibility, with a twofold change in the MIC. Our result was consistent with Linkevicius’s report showing that defects in LPS-related genes (*rfaC*, *rfaE*, *lpcA*) could cause low levels of TGC resistance (Linkevicius et al., 2016).

In our study, the Insertion sequences played an important role in phenotypic changes in antibiotic resistance and genomic variation during within-host evolution, causing gene devitalization and duplication. Antibiotic resistance caused by ISs has been previously reported (Yang et al., 2020). Under long-term antibiotic stress, genomic variation caused by ISs could help the host overcome environmental challenges. Compared to XDX16 and XDX31, longer-term usage of the β-lactam-β-lactamase inhibitor and TGC led to the IS*Kpn14* insertion in *wbbl*, IS*Kpn26* insertion in *ramR* and replicative IS*26* transposition with the *bla*_KPC-2_ transposon structure. The ISs above were widely distributed in the genomes of the baseline strains, and their movement in the genome led to key antibiotic resistance. Furthermore, composite transposons with two identical ISs, such as IS*26* on the *bla*_KPC-2_-bearing plasmid in our study, can become mobilized and transferable among different strains. The dissemination of antibiotic resistance genes could cause great challenges to public health (Partridge et al., 2018). In the future, more clinical isolates would be studied to investigate the characteristic of CRKP that are prone to develop last line antibiotic resistance.

## Conclusions

In brief, we tracked how a CRKP strain developed TGC and CZA resistance phenotypes during within-host antibiotic treatment. Three different genomic mutations were identified in XDX51, and all of them were caused by Insertion sequences. Double copies of *bla*_KPC-2_ contributed to the CZA MIC changes due to enhanced ceftazidime hydrolysis. CZA resistance occurred without previous exposure to CZA. In addition, *ramR* inactivation led to TGC resistance. Furthermore, inactivation of *wbbL* was identified as being associated with LPS O-antigen deficiency and slightly reduced the susceptibility to TGC. The ST4496 clone needs attention, as it has the potential to evolve TGC and CZA resistance rapidly under TGC and β-lactam antibiotic exposure.

## Data Availability Statement

The datasets presented in this study can be found in online repositories. This Whole Genome Shotgun project has been deposited at GenBank under the accession JAIWPV000000000-JAIWPW000000000.

## Ethics Statement

Written informed consent was obtained from the individual(s) for the publication of any potentially identifiable images or data included in this article.

## Author Contributions

XHa and QS conceived the idea and designed the experiments. XHa and YM performed the experiments. XHa analyzed the data. JQ, PZ, and PL helped with materials and reagents. XHa wrote the manuscript. YJ, DZ, XW, XHu, and YY reviewed the manuscript. All authors contributed to the article and approved the submitted version.

## Funding

This work was supported by the China National Natural Science Foundation grant (81830069).

## Conflict of Interest

The authors declare that the research was conducted in the absence of any commercial or financial relationships that could be construed as a potential conflict of interest.

## Publisher’s Note

All claims expressed in this article are solely those of the authors and do not necessarily represent those of their affiliated organizations, or those of the publisher, the editors and the reviewers. Any product that may be evaluated in this article, or claim that may be made by its manufacturer, is not guaranteed or endorsed by the publisher.

## References

[B1] Antinori E. Unali I. Bertoncelli A. Mazzariol A. (2020). Klebsiella Pneumoniae Carbapenemase (KPC) Producer Resistant to Ceftazidime–Avibactam Due to a Deletion in the Blakpc3 Gene. Clin. Microbiol. Infect. 26, 946.e1–946.e3. doi: 10.1016/j.cmi.2020.02.007 32061796

[B2] Anton D. N. (1995). Resistance to Mecillinam Produced by the Co-Operative Action of Mutations Affecting Lipopolysaccharide, Spot, and Cya or Crp Genes of Salmonella Typhimurium Typhimurium. Mol. Microbiol. 16, 587–595. doi: 10.1111/j.1365-2958.1995.tb02421.x 7565117

[B3] Barnes M. D. Winkler M. L. Taracila M. A. Page M. G. Desarbre E. Kreiswirth E. B. N. (2017). Klebsiella Pneumoniae Carbapenemase-2 (KPC-2), Substitutions at Ambler Position Asp179, and Resistance to Ceftazidime- Avibactam: Unique Antibiotic-Resistant Phenotypes Emerge From ␤-Lactamase Protein Engineering Melissa. MBio 2, 1–17. doi: 10.1128/mBio.00528-17 PMC5666153 29089425

[B4] Barrick D. E. Deatherage J. E. (2014). Identification of Mutations in Laboratory Evolved Microbes From Next-Generation Sequencing Data Using Breseq. Methods Mol. Biol. 1151, 1–22. doi: 10.1007/978-1-4939-0554-6 PMC4239701 24838886

[B5] Barton B. M. Harding G. P. Zuccarelli A. J. (1995). A General Method for Detecting and Sizing Large Plasmids. Anal. Biochem. 226, 235–240. doi: 10.1006/abio.1995.1220 7793624

[B6] Davis M. R. Goldberg J. B. (2012). Purification and Visualization of Lipopolysaccharide From Gram-Negative Bacteria by Hot Aqueous-Phenol Extraction. J. Vis. Exp. 1, 3196. doi: 10.3791/3916 PMC3466933 22688346

[B7] Duin C. D. Infectious D. Carolina N. Farm M. Kaye K. S. Kalayjian R. C.. (2014). Tigecycline Therapy for Carbapenem-Resistant Klebsiella Pneumoniae ( CRKP ) Bacteriuria Leads to Tigecycline Resistance. Clin. Microbiol. Infect. 20, O1117–O1120. doi: 10.1111/1469-0691.12714 24931918 PMC4265572

[B8] Erbing C. Lindberg B. Lönngren J. (1977). Structural Studies on the Klebsiella O Group 12 Lipopolysaccharide. Carbohydr. Res. 56, 1–5. 902268 10.1016/s0008-6215(00)83359-5

[B9] Follador R. Heinz E. Wyres K. L. Ellington M. J. Kowarik M. Holt K. E.. (2016). The Diversity of Klebsiella Pneumoniae Surface Polysaccharides. Microb. Genomics 2, e000073. doi: 10.1099/mgen.0.000073 PMC5320592 28348868

[B10] Galani I. Antoniadou A. Karaiskos I. Kontopoulou K. Giamarellou H. Souli M. (2019). Genomic Characterization of a KPC-23-Producing Klebsiella Pneumoniae ST258 Clinical Isolate Resistant to Ceftazidime-Avibactam. Clin. Microbiol. Infect. 25, 763.e5–763.e8. doi: 10.1016/j.cmi.2019.03.011 30928562

[B11] Hentschke M. Wolters M. Sobottka I. Rohde H. Aepfelbacher M. (2010). ramR Mutations in Clinical Isolates of Klebsiella Pneumoniae With Reduced Susceptibility to Tigecycline. Antimicrob. Agents Chemother. 54, 2720–2723. doi: 10.1128/AAC.00085-10 20350947 PMC2876394

[B12] Holt K. E. Wertheim H. Zadoks R. N. Baker S. Whitehouse C. A. Dance D. (2015). Genomic Analysis of Diversity, Population Structure, Virulence, and Antimicrobial Resistance in Klebsiella Pneumoniae, an Urgent Threat to Public Health. Proc. Natl. Acad. Sci. U.S.A. 112, E3574–E3581. doi: 10.1073/pnas.1501049112 26100894 PMC4500264

[B13] Huang T. W. Lam I. Chang H. Y. Tsai S. F. Palsson B. O. Charusanti P. (2014). Capsule Deletion *via* a λ-Red Knockout System Perturbs Biofilm Formation and Fimbriae Expression in Klebsiella Pneumoniae MGH 78578. BMC Res. Notes 7, 1–13. doi: 10.1186/1756-0500-7-13 24398052 PMC3892127

[B14] Humphries R. M. Hemarajata P. (2017). Resistance to Ceftazidime-Avibactam in Klebsiella Pneumoniae Due to Porin Mutations & the Increased Expression of KPC-3. Antimicrob. Agents Chemother. 61, e00537–e00517. doi: 10.1128/AAC.00537-17 28396547 PMC5444130

[B15] Izquierdo L. Merino S. Regue M. Rodriguez F. Toma J. M. (2003). Synthesis of a Klebsiella Pneumoniae O-Antigen Heteropolysaccharide ( O12 ) Requires an ABC 2 Transporter. J. Bacteriol. 185, 1634–1641. doi: 10.1128/JB.185.5.1634-1641.2003 12591881 PMC148082

[B16] Koren S. Walenz B. P. Berlin K. Miller J. R. Bergman N. H. Phillippy A. M. (2017). Canu : Scalable and Accurate Long-Read Assembly *via* Adaptive K-Mer Weighting and Repeat Separation. Genome Res. 27, 722–736. doi: 10.1101/gr.215087.116 28298431 PMC5411767

[B17] Leth K. Katrine N. Hansen H. Boye J. Jenny N. Knudsen D.. (2019). Mutational Change of CTX - M - 15 to CTX - M - 127 Resulting in Mecillinam Resistant Escherichia Coli During Pivmecillinam Treatment of a Patient. Microbiologyopen 8, e941. doi: 10.1002/mbo3.941 31573735 PMC6925186

[B18] Lin Y. Huang Y. Huang H. Yang T. Wang F. (2016). International Journal of Antimicrobial Agents *In Vivo* Evolution of Tigecycline-Non-Susceptible Klebsiella Pneumoniae Strains in Patients: Relationship Between Virulence and Resistance. Int. J. Antimicrob. Agents 48, 485–491. doi: 10.1016/j.ijantimicag.2016.07.008 27575728

[B19] Linkevicius M. Anderssen J. M. Sandegren L. Andersson D. I. (2016). Fitness of Escherichia Coli Mutants With Reduced Susceptibility to Tigecycline. J. Antimicrob. Chemother. 71, 1307–1313. doi: 10.1093/jac/dkv486 26851608 PMC4830415

[B20] Livak K. J. Schmittgen T. D. (2001). Analysis of Relative Gene Expression Data Using Real-Time Quantitative PCR and the 2-ΔΔCT Method. Methods 25, 402–408. doi: 10.1006/meth.2001.1262 11846609

[B21] Livermore D. M. Maya J. J. Nordmann P. Wang H. Woodford N. Quinn J. P. (2015a). Clinical Epidemiology of the Global Expansion of Klebsiella Pneumoniae Carbapenemases. Lancet Infect. Dis. 13, 785–796. doi: 10.1016/S1473-3099(13)70190-7.Clinical PMC4673667 23969216

[B22] Livermore D. M. Warner M. Jamrozy D. Mushtaq S. Nichols W. W. Mustafa N.. (2015b). *In Vitro* Selection of Ceftazidime-Avibactam Resistance in Enterobacteriaceae With KPC-3 Carbapenemase. Antimicrob. Agents Chemother. 59, 5324–5330. doi: 10.1128/AAC.00678-15 26100712 PMC4538485

[B23] Moradigaravand D. Martin V. Peacock S. J. Parkhill J. (2017). Evolution and Epidemiology of Multidrug-Resistant Klebsiella Pneumoniae in the United Kingdom and Ireland. MBio 8, 1–13. doi: 10.1128/mBio.01976-16 PMC5358916 28223459

[B24] Partridge S. R. Kwong S. M. Firth N. Jensen S. O. (2018). Mobile Genetic Elements Associated With Antimicrobial Resistance. Clin. Microbiol. Rev. 31, e00088–e00017. doi: 10.1128/CMR.00088-17 30068738 PMC6148190

[B25] Rice L. B. (2008). Federal Funding for the Study of Antimicrobial Resistance in Nosocomial Pathogens : No ESKAPE. J. Infect. Dis. 197, 1079–1081. doi: 10.1086/533452 18419525

[B26] Salehi B. Ghalavand Z. Yadegar A. Eslami G. (2021). Characteristics and Diversity of Mutations in Regulatory Genes of Resistance-Nodulation-Cell Division Efflux Pumps in Association With Drug-Resistant Clinical Isolates of Acinetobacter Baumannii. Antimicrob. Resist. Infect. Control 10, 1–12. doi: 10.1186/s13756-021-00924-9 33691788 PMC7944621

[B27] Seemann T. (2014). Prokka : Rapid Prokaryotic Genome Annotation. Bioinformatics 30, 2068–2069. doi: 10.1093/bioinformatics/btu153 24642063

[B28] Shen Z. Ding B. Ye M. Wang P. Bi Y. Wu S.. (2017). High Ceftazidime Hydrolysis Activity and Porin OmpK35 Deficiency Contribute to the Decreased Susceptibility to Ceftazidime/Avibactam in KPC-Producing Klebsiella Pneumoniae. J. Antimicrob. Chemother. 72, 1930–1936. doi: 10.1093/jac/dkx066 28333323

[B29] Shen P. Wei Z. Jiang Y. Du X. Ji S. Yu Y.. (2009). Novel Genetic Environment of the Carbapenem-Hydrolyzing Beta-Lactamase KPC-2 Among Enterobacteriaceae in China. Antimicrob. Agents Chemother. 53, 4333–4338. doi: 10.1128/AAC.00260-09 19620332 PMC2764158

[B30] Shields R. K. Chen L. Cheng S. Chavda K. D. Press E. G. (2017). Emergence of Ceftazidime-Avibactam Resistance Due to Plasmid-Borne blaKPC-3 Mutations During Treatment of Carbapenem-Resistant Klebsiella Pneumoniae Infections. Antimicrob. Agents Chemother. 61, 1–11. 10.1128/AAC.02097-16 PMC5328542 28031201

[B31] Shirley M. (2018). Ceftazidime-Avibactam: A Review in the Treatment of Serious Gram-Negative Bacterial Infections. Drugs 78, 675–692. doi: 10.1007/s40265-018-0902-x 29671219

[B32] Shrivastava S. R. Shrivastava P. S. Ramasamy J. (2018). World Health Organization Global Priority List of Antibiotic-Resistant Bacteria to Guide Research, Discovery, and Development of New Antibiotics. J. Med. Soc. 32, 76–77. doi: 10.4103/jms.jms_25_17

[B33] Spiliopoulou I. Kazmierczak K. Stone G. G. (2020). *In Vitro* Activity of Ceftazidime/Avibactam Against Isolates of Carbapenem-Non-Susceptible Enterobacteriaceae Collected During the INFORM Global Surveillance Programm -17. J. Antimicrob. Chemother. 75, 384–391. doi: 10.1093/jac/dkz456 PMC6966093 31742604

[B34] Ullmann U. (1998). Klebsiella Spp. As Nosocomial Pathogens : Epidemiology, Taxonomy, Typing Methods, and Pathogenicity Factors. Clin. Microbiol. Rev. 11, 589–603. doi: 10.1128/CMR.11.4.589 9767057 PMC88898

[B35] Van Duin D. Lok J. J. Earley M. Cober E. Richter S. S. Perez F.. (2018). Colistin *Versus* Ceftazidime-Avibactam in the Treatment of Infections Due to Carbapenem-Resistant Enterobacteriaceae. Clin. Infect. Dis. 66, 163–171. doi: 10.1093/cid/cix783 29020404 PMC5850032

[B36] Veleba M. Schneiders T. (2012). Tigecycline Resistance Can Occur Independently of the ramA Gene in Klebsiella Pneumoniae. Antimicrob. Agents Chemother. 56, 4466–4467. doi: 10.1128/AAC.06224-11 22644034 PMC3421586

[B37] Villa L. Feudi C. Fortini D. García-fernández A. Carattoli A. (2014). Genomics of KPC-Producing Klebsiella Pneumoniae Sequence Type 512 Clone Highlights the Role of RamR and Ribosomal S10 Protein Mutations in Conferring Tigecycline Resistance. Antimicrob. Agents Chemother. 58, 1707–1712. doi: 10.1128/AAC.01803-13 24379204 PMC3957836

[B38] Yamasaki S. Nikaido E. Nakashima R. Sakurai K. Fujiwara D. Fujii I.. (2013). The Crystal Structure of Multidrug-Resistance Regulator RamR With Multiple Drugs. Nat. Commun. 4, 2078. doi: 10.1038/ncomms3078 23800819

[B39] Yang T. Y. Wang S. F. Lin J. E. Griffith B. T. S. Lian S. H. Hong Z.. (2020). Contributions of Insertion Sequences Conferring Colistin Resistance in Klebsiella Pneumoniae. Int. J. Antimicrob. Agents 55, 105894. doi: 10.1016/j.ijantimicag.2020.105894 31923567

[B40] Ye M. Ding B. Qian H. Xu Q. Jiang J. Huang J.. (2017). *In Vivo* Development of Tigecycline Resistance in Klebsiella Pneumoniae Owing to Deletion of the ramR Ribosomal Binding Site. Int. J. Antimicrob. Agents 50, 523–528. doi: 10.1016/J.IJANTIMICAG.2017.04.024 28668690

[B41] Zankari E. Hasman H. Cosentino S. Vestergaard M. Rasmussen S. Lund O.. (2012). Identification of Acquired Antimicrobial Resistance Genes. J. Antimicrob. Chemother. 67, 2640–2644. doi: 10.1093/jac/dks261 22782487 PMC3468078

[B42] Zhang P. Shi Q. Hu H. Hong B. Wu X. Du X.. (2020). Emergence of Ceftazidime/Avibactam Resistance in Carbapenem-Resistant Klebsiella Pneumoniae in China. Clin. Microbiol. Infect. 26, 124.e1–124.e4. doi: 10.1016/j.cmi.2019.08.020 31494252

