# Supplementary material for: Emergence of Ceftazidime/Avibactam and Tigecycline Resistance in Carbapenem-Resistant Klebsiella pneumoniae Due to In-Host Microevolution
Source: Front Cell Infect Microbiol. 2021 Oct 25;11:757470. doi: 10.3389/fcimb.2021.757470 (PMC8573091; doi:10.3389/fcimb.2021.757470)
Supplement: Supplementary file 1 [file DataSheet_1.docx]

Table S1 Primers in our study

| Name | Primer sequence（5’- 3’）^*^ | | Use | |
| --- | --- | --- | --- | --- |
| *wbbL*_conf_F | ATCCGGAGATAGGGATTGACTGG | *wbbL* mutation confirmation | |  |
| *wbbL*_conf_R | ATGACAGCGGTCATATTCTGCCA |  |  |  |
| PROKKA_02802-F^a^ | GCCTTTGCCGTCTACCCTTT | SNPs confirmation | |  |
| PROKKA_02802-R^a^ | AGTACCGCGAGGCTTTTGAT |  | |  |
| fbaA_2-F^b^ | AATTAACGCCGTGCGATGAC |  | |  |
| fbaA_2-R^b^ | GGTTATTACCCATTGGGATCACG |  | |  |
| PROKKA_03128-F^c^ | TTGCACGGAAAAGAGCCCTA |  | |  |
| PROKKA_03128-R^c^ | GCAGAAGGCATCCTTTAGTGG |  | |  |
| *wbbL*_ko_F | CACAAGTGGCTATTTAAAAATAATAATAAAAAATTTATCCTAAAGGTAATCGATTAATGattccggggatccgtcgacc | *wbbL* knockout | |  |
| *wbbL*_ko_R | GGGATGCGCTCCGGATGAATATGATGATCTCATATCAGGAACTTGTTCGCACCTTCCTTAtgtaggctggagctgcttc |  |  |  |
| *wbbL*_koconf_F | GACTGGCCGGCGCTGGGCGA | *wbbL* knockout confirmation | |  |
| *wbbL*_koconf_R | GACTGGCCGGCGCTGGGCGA |  | |  |
| *wbbL*_Pcr2.1_F | GATATCCATCACACTGGCGGCCGCTCGAGCATGCATCTAGcagtcagagcgaagcatcat | *wbbL* complementation | |  |
| *wbbL*_Pcr2.1_R | TTGTAATACGACTCACTATAGGGCGAATTGGGCCCTCTAGcagggataatcgggcaaaca |  |  |  |
| *bla*_KPC-2_-F | GCGGCAGCAGTTTGTTGATT | qRT-PCR | |  |
| *bla*_KPC-2_-R | CGGCATAGTCATTTGCCGTG |  |  |  |
| *acrA*-F | TACCGCAACAGGGTGTTACC |  |  |  |
| *acrA*-R | CTTCCTGCGCTTTTACCTGC |  |  |  |
| *acrB*-F | CGACTCGACGTTCTGCTTCT |  |  |  |
| *acrB*-R | TGCGCTGGATGCTGATCTAC |  |  |  |
| *ramA*-F | GCATCAACCGCTGCGTATT |  |  |  |
| *ramA*-R | GGGTAAAGGTCTGTTGCGAAT |  |  |  |
| *bla*_KPC-2_-c-F | GCGGCAGCAGTTTGTTGATT |  |  |  |
| *bla*_KPC-2_-c-R | CGGCATAGTCATTTGCCGTG |  |  |  |
| *rpoB*-F | TGAACAAGCTGGATTCGCCT |  |  |  |
| *rpoB*-R | CGCGCAGACCAACGAATATG |  |  |  |

* Capital letters indicate sequences for recombination, lowercase letters indicate primers for amplification in gene knockout and complementation strains

Table S2 SNPs between XDX51 and XDX16^*^

| Positions in  XDX16 genome | SNPs | annotation | gene | description |
| --- | --- | --- | --- | --- |
| 2907019 bp | T➝G | L355R (CTG➝CGG) | *PROKKA_02802* | Citrate transporter |
| 3248841bp | T➝C | A208A (GCA➝GCG) | *fbaA_2* | Putative fructose-bisphosphate  aldolase |
| 3252845 bp | T➝A | L63F (TTA➝TTT) | *PROKKA_03128* | D-arabitol-phosphate  dehydrogenase |

*: SNPs between XDX51 and the wild type XDX16 were detected using Breseq. The wild type XDX31 had the same SNPs as XDX51 compared to XDX16, indicating the SNPs between XDX51 and XDX16 do not contribute to antibiotic susceptibility.


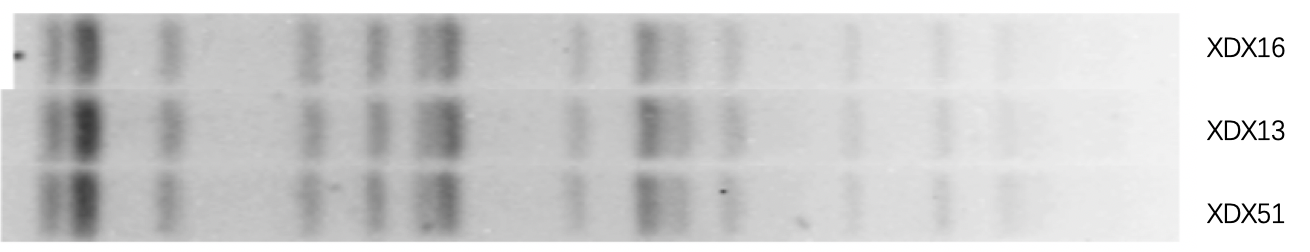


Figure S1 PFGE patterns. XDX13 was another tigecycline sensitive CRKP isolated from the same patient as XDX16 and XDX51. The series of CRKP strains had the same PFGE pattern.


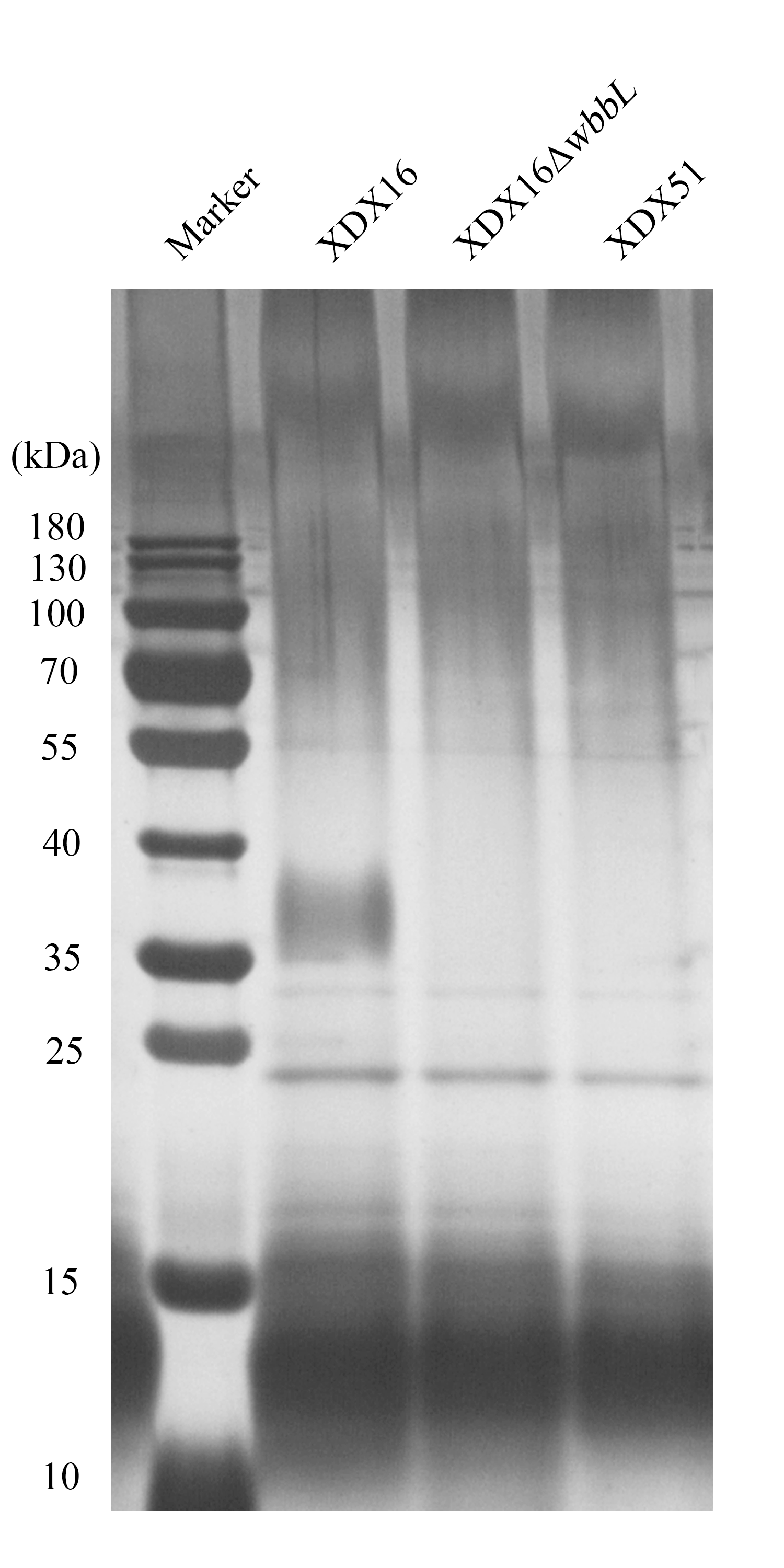


Figure S2. LPS profiles of *wbbL* wild-type and mutant strains. M: marker. In *wbbL* mutant strains, O-antigen bands from 35 to 40 kDa was missing.
